# Potassium Channel Activation Is Involved in the Cardiovascular Effects Induced by Freeze Dried* Syzygium jambolanum* (Lam.) DC Fruit Juice

**DOI:** 10.1155/2018/4827461

**Published:** 2018-10-08

**Authors:** Kívia S. Assis, Islania G. A. Araújo, Fátima de L. A. A. de Azevedo, Priscilla M. P. Maciel, Natália T. Machado Calzerra, Tays A. F. da Silva, Valéria L. Assis, Aliny P. de Vasconcelos, Carlos A. G. Santos, Bruno R. L. A. Meireles, Angela M. T. M. Cordeiro, Demetrius A. M. Araújo, Thais P. Ribeiro, Isac A. Medeiros

**Affiliations:** ^1^Programa de Pós-Graduação em Produtos Naturais e Sintéticos Bioativos/Centro de Ciências da Saúde (CCS)/Universidade Federal da Paraíba (UFPB), João Pessoa 58059-900, Brazil; ^2^Departamento de Ciências Farmacêuticas, (CCS)/Universidade Federal da Paraíba (UFPB), João Pessoa 58059-900, Brazil; ^3^Departamento de Biotecnologia, Centro de Biotecnologia/Universidade Federal da Paraíba (UFPB), João Pessoa 58059-900, Brazil; ^4^Centro de Educação e Saúde, Unidade Acadêmica de Biologia e Química, Universidade Federal de Campina Grande (UFCG) Cuité, Paraíba, 58750-000, Brazil; ^5^Departamento de Tecnologia de Alimentos, Centro de Tecnologia e Desenvolvimento Regional - CTDR, Universidade Federal de Paraíba, João Pessoa, Paraíba, Brazil

## Abstract

This work aimed to explore the cardiovascular effects induced by freeze-dried juice from Syzygium jambolanum (Lam.) DC fruits (JSJ). JSJ presented high polyphenol content and steroids. HPLC analysis revealed that 2,5-dihydroxybenzoic and caffeic acid were present in higher amounts in the JSJ extract. In rat, JSJ induces hypotension and vasodilatation in mesenteric arteries, with or without vascular endothelium. JSJ-mediated vasodilation response against contractions induced with KCl (60 mM) depolarizing solution was significantly lower than the responses induced by JSJ when evaluated against phenylephrine-induced contractions. To investigate the involvement of potassium channels we used Tyrode's solution with KCl (20 mM) or tetraethylammonium (1.0, 3.0, or 5.0 mM). In these conditions JSJ-induced effects were significantly attenuated. To investigate the potassium channel subtypes involved in the response, we used 4-aminopyridine, glibenclamide, BaCl_2_, and iberiotoxin. In the presence (simultaneous) of different potassium channel blockers we observed a significant attenuation of JSJ-induced effect. Inhibition was also observed when using BaCl_2_, glibenclamide, or 4-aminopyridine, separately. However, incubation with iberiotoxin did not promote changes in either maximum effect, or potency. We also evidenced a discrete participation of Ca_V_ channels in the JSJ-induced vasorelaxant effect. In addition, patch-clamp studies demonstrated that JSJ could activate potassium channels. In conclusion, JSJ promotes hypotension and vasorelaxation in rats, involving, at least, the activation of potassium channels.

## 1. Introduction

Fruits and vegetables are generally rich sources of vitamins, minerals, and fiber. They contain a variety of bioactive compounds and have been recognized as nutritionally important foods in the human diet [1]. Among their bioactive substances, phenol compounds have recently received great attention due to their antioxidant capacity and beneficial effects on human health, being useful in the prevention and treatment of cancer, neurodegenerative disease, cardiovascular disease, and other pathologies [2].

*Syzygium jambolanum* (Lam.) DC, a tree popularly known in Brazil as* jambu* or* jambolão*, is native to the tropics, particularly India, Thailand, the Philippines, and Madagascar. The plant has been introduced to many tropical regions such as eastern and western Africa, and also Brazil. It is found in various regions or states of the southeast, south, north and northeast [3].

The major acid present in the fruit of* Syzygium jambolanum* (Lam.) DC is malic acid, with traces of oxalic acid, gallic acid, and tannins that confer a characteristic astringency to the fruit. It is rich in minerals such as potassium, calcium, phosphorus, iron, and zinc; water-soluble vitamins such as ascorbic acid, thiamine and niacin; and carbohydrates such as glucose, mannose, sucrose, maltose, fructose and galactose. Preliminary phytochemical analyses have revealed simple heteroside phenols, flavonoids, alkaloids, and iridoids [3–6].

For many years,* Jambolão* has been used in traditional indigenous medicine to treat diseases such as diabetes mellitus and as a bactericide. The literature reports hypoglycemic, hypotensive, diuretic, cardiotonic, astringent, anti-inflammatory, antioxidant, neuroprotective, antipyretic, anticonvulsant, anti-hemorrhagic, antiulcer, hepatoprotective, carminative, and radio-protective properties [4, 5].

Studies performed with the cardiovascular system and* Syzygium jambolanum* (Lam.) DC hydroalcoholic dried leaf extract have demonstrated hypotensive activity in normotensive rats, and its chloroform fraction presents vasorelaxant effect in mesenteric rat artery rings (when pre-contracted with calcium) [7]. More recently, in spontaneously hypertensive rats, (submitted to treatment for 8 weeks with the same extract at 0.5 g/kg/day) the group showed dose-dependent reductions in blood pressure and heart rate [8]. In both studies, the authors suggested that the extract might contain phenolic compounds capable of non-competitive blocking of type L calcium channels [3].

Studies performed with* Syzygium jambolanum* (Lam.) DC hydroalcoholic fruit extracts also demonstrate hypotensive activity in nonanesthetized normotensive rats, as well as a vasorelaxant effect in rat superior mesenteric artery rings, an endothelium dependent effect [9]. Still, little is known concerning the action of this juice on the cardiovascular system or its mechanism of action.

This study aimed to evaluate* in vivo* and* in vitro* cardiovascular effects induced in rats using freeze dried* Syzygium jambolanum* (Lam.) DC fruit juice (JSJ). We sought to elucidate the mechanisms involved and to provide new information towards development of effective, safe, and low cost alternative therapies.

## 2. Materials and Methods

### 2.1. Laboratory Animals and Ethics Statement

Wistar rats (*Rattus norvegicus*) were used, weighing between 250 and 300g, from the* Biotério Prof. George Thomas*, of Federal University of Paraíba (UFPB), and kept under controlled temperature conditions (21 ± 1°C), light-dark cycle of 12 hours (6am–6pm), and free access to water and feed (Purina®). All protocols were approved by the UFPB Ethics Committee on Animal Use with certificate N^o.^ 1405/13.

### 2.2. Chemicals

The following substances were used in all protocols: L (-) phenylephrine hydrochloride (Phe), acetylcholine hydrochloride (ACh), tetraethylammonium bromide (TEA), glibenclamide, 4-aminopyridine (4-AP), iberiotoxin, barium chloride dihydrate (BaCl_2_), acetonitrile, formic acid, Ciocalteu reagent, gallic acid, and di(phenyl)-(2,4,6-trinitrophenyl) iminoazanium (DPPH), obtained from Sigma-Aldrich Brasil Ltda (São Paulo-SP, Brazil), with sodium heparin from the Roche Chemical Company (Rio de Janeiro, Brazil), and sodium thiopental from Cristália (São Paulo, SP, Brazil). All chemicals used in the experiments were of analytical reagent grade.

### 2.3. Sample Preparation and Phytochemical Screening

To obtain freeze dried* Syzygium jambolanum* (Lam.) DC juice, fresh fruit was collected in the municipality of Marcação, PB, Brazil (06°46′10.9′′S 35°01′07.0′′W). A voucher specimen of* Syzygium jambolanum* (Lam.) DC was deposited in the “Herbário do Centro de Educação e Saúde” Herbarium of the Federal University of Campina Grande, N^o.^ 228. The seeds were removed, and the pulp together with the bark was triturated (Philips Juicer Silver RI1858 - 650W); the liquid was sonicated for 16 minutes and centrifuged at 15°C at 4800 rpm for 1 hour. The supernatant was frozen at −80°C for 24 hours and then freeze-dried at −40°C under a pressure of 0.024 mbar for 48 hours, then frozen at −20°C until the day of the experiment. Then, JSJ was subjected to a preliminary phytochemical screening to detect secondary metabolites through chemical characterization reactions (coloration and/or precipitation) to classify the substances [10, 11]. The secondary metabolites investigated were tannins, flavonoids, alkaloids, and steroidal saponins.

### 2.4. JSJ Analysis Using High Performance Liquid Chromatography

HPLC analysis was performed on a Shimadzu high-performance liquid chromatograph (Kyoto, Japan), equipped with a Rheodyne 7125i automatic injector and a UV / VIS detector. Columns used were a Shimadzu LC-18 (25cm x 4.6mm, 5 *μ*m particle size, Supelco, Bellefonte, PA) column and a C-18 ODS Shimadzu pre-column. For the identification of phenolic compounds, the sample was eluted with a gradient system consisting of solvent A (2% acetic acid, v/v) and solvent B (acetonitrile: methanol, 2: 1, v/v) with a flow rate of 1 mL/min. The column temperature was maintained at 25°C and the injection volume was 20 *μ*l. The gradient system was started from 90% A to 0 min, 88% A in 3 min, 85% A in 6 min, 82% A in 10 min, 80% A in 12 min, 70% A in 15 min, 65% A in 20 min, 60% A in 25 min, 50% A in 30-40 min, 75% A in 42 min and 90% A in 44 min [12]. The total chromatographic run was 50 minutes. Peaks of the phenolic compounds were monitored at 280 nm. LabSsolutions (Shimadzu) software was used to control the LC-UV system and data processing. Phenolic compounds were identified by comparing the retention times with phenolic compound standards and were quantified at concentrations of mg / 100 g from calibration curves and the chromatogram was recorded in the LabSolutions Data System software.

### 2.5. Total Phenol Content

The total JSJ phenolic content was determined in triplicate and expressed in gallic acid mg equivalents (GAE) using the method (with modifications) described by Slinkard and Singleton [13], and colorimetric Folin Ciocalteu reagent. JSJ (300 *μ*l), 60 *μ*L of Folin Ciocalteu reagent, and 2.460 *μ*L of Milli-Q water were mixed in a vortex and incubated for 1 min before adding 180 *μ*l of Na_2_CO_3_ solution at 15%. This mixture was then left to stand for 120 minutes at ambient temperature, and the absorbance was measured at 760 nm.

### 2.6. Antioxidant Activity

JSJ antioxidant activity was determined by tests with DPPH● in accordance with Brand-Williams, Cuvelier and Berset (1995) [14], with modifications. An alcoholic solution containing 0.006 mM of DPPH was prepared and to appropriately labeled test tubes JSJ was added, then 2,700 *μ*L of DPPH solution and an adequate volume of ethanol were added to yield samples of JSJ mixed at varying volumes (150, 180, 210, and 240 *μ*L), resulting in different concentrations (250, 300, 350 and 400 *μ*g/mL, respectively). The control consisted of ethanol (300 *μ*L) and 2,700 *μ*L of DPPH solution. The 3 mL of solution in test tubes reacted in an ultrasonic bath for 30 minutes under light. The absorbance of the DPPH and JSJ solutions was then taken in a UV-Vis spectrophotometer (Shimadzu UV-2550, Kyoto, Japan) at 517 nm. Antioxidant capacity was expressed as the concentration of antioxidant required to reduce the original amount of free radicals by 50% (EC_50_), and the values expressed in *μ*g/mL of JSJ/DPPH [14].

### 2.7. Direct Measurement of Intra-Aortic Pressure in Non-Anesthetized Rats

Intra-aortic pressure was recorded using a technique described by Dantas et al. [15] In rats anesthetized with sodium thiopental (45 mg/kg, i.v.), the lower abdominal aorta and inferior vena cava were cannulated via the left femoral artery and vein using polyethylene catheters. The catheters were subsequently filled with heparinized saline solution and tunneled under the skin to emerge between the shoulder blades. Blood pressure was measured 24 h after surgery by connecting the arterial catheter to a precalibrated pressure transducer (MLT0380/D, ADInstruments, Australia) and connected to a data acquisition system (ADInstruments PowerLab, Unit 13, 22 Lexington Avenue, Bella Vista, NSW, Australia). The software for acquisition and data analysis was LabChart version 5.0. Data were also displayed at 2000 Hz. From the pressure of each pulse, the computer calculated the mean arterial pressure and heart rate. The venous catheter was used for drug administration, and after stabilization of the cardiovascular parameters, varied doses of JSJ were randomly administered (5, 10, 30, 50, and 100 mg/kg i.v.).

### 2.8. Vascular Reactivity in Superior Mesenteric Rat Artery Rings

The rats were euthanized and the superior mesenteric artery was removed. It was cleaned of adherent connective tissues and then sliced into rings (1-2 mm) in a Tyrode solution containing the following composition (mM): NaCl 138.16, KCl 4.0, CaCl_2_ 2.0, MgCl_2_ 1.05, NaH_2_PO_4_ 0.42, NaHCO_3_ 10.0, and glucose 5.6 (pH = 7.4); maintained at 37°C and gasified with a carbogenic mixture (95% O_2_, and 5% CO_2_). The preparations were stabilized at a resting tension of 0.75 g for one hour as determined beforehand by length-tension relationship experiments. During stabilization, the solution was replaced every 15 min to avoid metabolite accumulation. The contraction force was recorded isometrically on a force transducer (MLT020, ADInstruments, Australia) connected to a data acquisition system (ML870/P, using LabChart version 7.0, ADInstruments, Australia). As needed, the endothelium was removed by gently rubbing the intimal surface of the vessels.

Endothelial integrity was qualitatively evaluated from degree of relaxation using ACh (10 *μ*M) while under the contractive activity effect induced by Phe (10 *μ*M). The rings were considered as denuded of endothelium when the relaxation effect induced by acetylcholine was lower than 10% and endothelium intact when the relaxation effect was above 90%.

The JSJ vasorelaxant effect was initially observed against continuing Phe (1 *μ*M) contraction, and while under this contraction tonus, increasing and cumulative concentrations of JSJ (10 - 5000 *μ*g/mL) were added. This occurred in rings with functional endothelium as well as those without it. The second set of experiments, evaluated the vasorelaxant effect of JSJ in the rings in the absence of functional endothelium; against contraction with a depolarizing KCl solution (60 mM).

To assess the involvement of K^+^ channels in the JSJ induced effect, we used Tyrode's solution modified with 20 mM KCl. The increase of external K^+^ concentration from 4 mM to 20 mM is sufficient to partially prevent K^+^ efflux and attenuate vasorelaxation as mediated by K^+^ channel opening [16, 17]. To discover which potassium channels might be involved in this effect, we used different pharmacological tools: TEA (1, 3, and 5 mM), BaCl_2_ (30 *μ*M), iberiotoxin (100 nM), glibenclamide (10 *μ*M), and 4-AP (1 mM) before the rings were contracted with Phe.

In addition, to evaluating the participation of potassium channels in the vasorelaxant effect induced by JSJ, we also investigated its effect on concentrations induced by CaCl_2_. The preparations were washed in Tyrode's solution (nominally without Ca^2+^), and the rings were then exposed to a depolarizing solution with 60 mM KCl (nominally without Ca^2+^); to obtain a cumulative concentration-response curve by sequentially adding CaCl_2_ (10^−6^ - 3x10^-2 ^M) to the medium. The process was repeated again, such that isolated concentrations of JSJ (3000 *μ*g/mL and 5000 *μ*g/mL) were incubated in preparations together with 60 mM KCl depolarizing solution (nominally without Ca^2+^), and the second concentration response curve was obtained.

### 2.9. Electrophysiological Recording

#### 2.9.1. Preparation of Vascular Smooth Muscle Cells

The mesenteric myocytes were enzymatically isolated from the Wistar rats by a procedure similar to that previously described by Pereira et al. [18]. Summarizing, the mesenteric vessel was removed and cleaned of all connective and fat tissues in cold physiological saline solution (PSS), containing (in mM): 137 NaCl, 5.6 KCl, 0.44 NaH_2_PO_4_, 0.42 Na_2_HPO_4_, 4.17 NaHCO_3_, 1.0 MgCl_2_, 2.6 CaCl_2_, 10 HEPES and 5 of glucose; the pH was adjusted to 7.4 with NaOH. To obtain mesenteric myocytes for electrophysiological evaluation, recently dissected tissues were cut lengthwise and then incubated at 37°C (for 30 min) in PSS, supplemented with 1 mg/ mL of bovine serum albumin (BSA), 0.7 mg/ mL of chymopapain, and 1.0 mg/ mL of dithiothreitol (DTT). The tissue was then submitted for 20 min to a low Ca^2+^ (0.05 mM CaCl_2_) PSS with an additional 1 mg/mL of BSA, 1 mg/ mL of collagenase type II, and 0.9 mg/mL of hyaluronidase. The individual cells were smoothly ground and acquired using a pipette and then aliquots of cell suspension were placed in an experimental chamber. The cells were maintained at ambient temperature (approximately 22-24°C) for at least 20 minutes, allowing adhesion to the glass-bottom of the chamber. The electrophysiological recordings were performed only in cells that under microscope exhibited the morphological characteristics of vascular smooth muscle cells (elongated and spindle-shaped).

#### 2.9.2. Whole-Cell Patch-Clamp Recording

Mesenteric myocyte cells were plated directly on glass slides and transferred to a recording chamber. The extracellular control solution contained (in mM) 145 NaCl, 5 KCl, 1.6 CaCl_2_, 1 MgCl_2_, 10 HEPES, 0.5 NaH_2_PO_4_, and 10 glucose; with a pH of 7.4, and an osmolarity of 0.3 osmol /l. Reticulation pipettes were filled with (in mM) 140 KCl, 10, EGTA, 1 MgCl_2_, and 5 glucose; the pH was adjusted to 7.2 with KOH, and an osmolarity of 0.3 osmol /L. The pipettes were removed from the glass capillaries (Perfecta, São Paulo, SP, Brazil) using a micropipette extractor (PC-10, Narishige, Japan). The pipettes had resistances of 3-4 MΩ when filled with pipette solution. We used Ag-AgCl wire as the reference electrode. An EPC-10 patch-clamp amplifier (HEKA Instruments, Germany), and pulse software were used to record the K^+^ currents in whole cells. The capacitive currents were compensated electronically, and a P/4 protocol was used to subtract linear flow and residual capacitance. The K^+^ currents were filtered at 3 kHz and sampled at 10 kHz. Cell membrane capacitance was measured automatically using an internal routine in the Pulse software (HEKA Instruments, Germany). The bath was continuously perfused at 1-2 mL /min throughout the entire experiment. The solutions were gravity fed to a solenoid valve which was mounted near the bath. The valve was used to select either of the two solutions. The individual current IK^+^ was generated by 200 ms depolarization pulses with a retention potential of from -60 mV to 60 mV. Myocyte cells current-voltage relationships were obtained using 200 ms depolarization pulses from -60 mV to 60 mV (in 10 mV increments) triggered every 5 seconds.

The data were collected after the configuration of whole cells was achieved and the current amplitude stabilized. Only cells with an input resistance of 1 GΩ were analyzed.

### 2.10. Statistical Analysis

Data were presented as mean ± SEM. The JSJ concentration-response curves were based on percentage relaxation of contractions induced by agonists. A value of 100% relaxation was assigned when the pre-treated rings returned to the base line voltage. The curves were adjusted using a variable tilt sigmoid fitting routine in GraphPad Prism® software, version 6.0 (GraphPad Software Inc., La Jolla, CA, USA). Maximum relaxation corresponded to maximum response (MR) for the highest concentration used. Pharmacological potency was determined as EC_50_ (substance inducing 50% of maximum effect). Statistical significance was determined by the non-paired Student's t test or “bidirectional” ANOVA, if appropriate after Bonferroni post-testing. P <0.05 were considered statistically significant.

The current recordings were fixed as pA/pF, and using FitMaster software (HEKA Instruments, Germany), data were extracted as mean ± SEM, of a number of cells (n = 7). The differences were statistically evaluated using Student's* t*-test. P <0.05 were considered statistically significant.

## 3. Results

### 3.1. Phytochemical Composition and Antioxidant Activity

**Figure 1:**
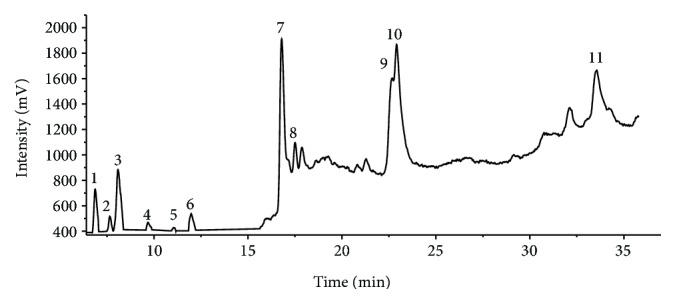
HPLC chromatogram of ethyl acetate fraction. Peaks: 1: catechin; 2: gentisic acid; 3: p-hydroxybenzoic acid; 4: vanillic acid; 5: syringic acid; 6: p-coumaric acid; 7: rutin; 8: myricetin; 9: caffeic acid; 10: quercetin; 11: chrysin.

Preliminary phytochemical analysis of JSJ revealed the presence of flavonoids and steroids. Determination of total phenolic content by the Folin-Ciocalteu method yielded levels of 988.55 ± 5.41 mg GAE/100g. JSJ antioxidant activity was expressed as EC_50_ (concentration JSJ needed to inhibit DPPH oxidation by 50%) at 302.95 ± 0.12 *μ*g/ml. HPLC of JSJ revealed the presence of 11 phenolic compounds (Figure 1) which were expressed in compound mg/100 g JSJ concentrations (Table 1).

### 3.2. Effect of JSJ on Blood Pressure

**Figure 2:**
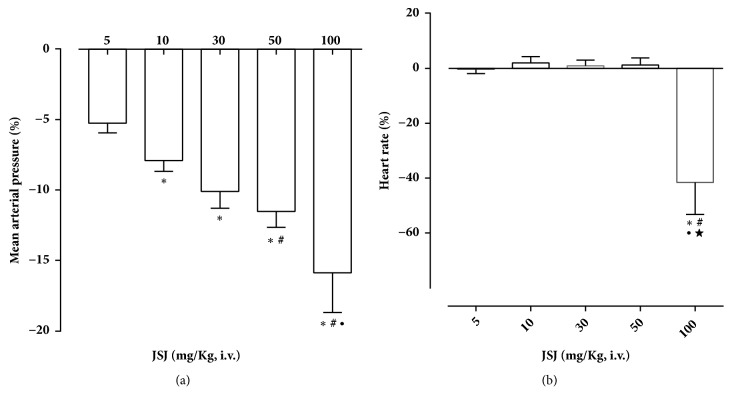
JSJ effect on mean arterial pressure (MAP) and heart rate (HR) of rats. Bar graph showing the changes in mean arterial pressure** (a)**, and heart rate** (b)** induced by the acute administration of JSJ (5, 10, 30, 50, and 100 mg/kg, randomly) in nonanesthetized rats. Values were mean ± SEM (n=5). We used “two-way” ANOVA following Bonferroni's post-test, *∗*p < 0.05 (vs. 5 mg/kg), #p < 0.05 (vs. 10 mg/kg), ∙p<0.05 (vs. 30 mg/kg), ★p<0.05 (vs. 50 mg/Kg).

Bolus administration of JSJ (5, 10, 30, 50 and 100 mg/kg) induced hypotension (respectively, -5.2 ± 0.6, -7.9 ± 0.7 -10.0 ± 1.2, -11.5 ± 1.1, -15.8 ± 2.8) (Figure 2(a)) and bradycardia was observed at the maximum dose of 100 mg/kg (-41.6 ± 11.6) (Figure 2(b)).

### 3.3. Vasorelaxant Effect Induced by JSJ in Rings Isolated from the Superior Mesenteric Artery of Rats

**Figure 3:**
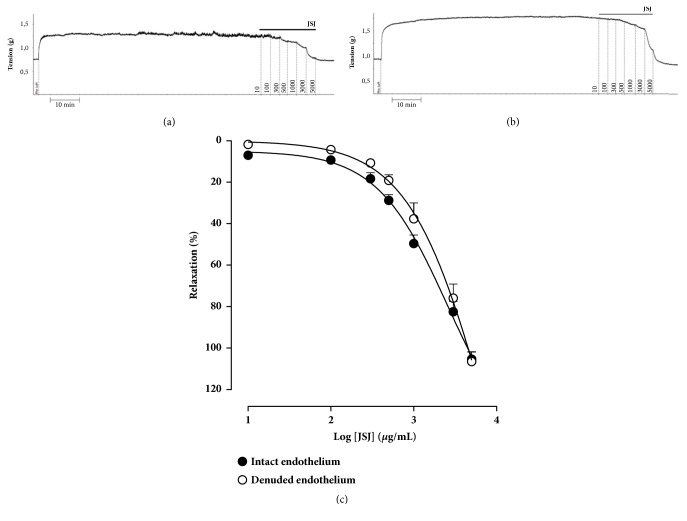
Vasorelaxant effect of JSJ in isolated rat mesenteric rings. Representative tracings showing vasodilator effect of JSJ in the presence (**a**) or absence (**b)** of functional endothelium. (**c**) Concentration-response curves to JSJ (10 - 5000 *μ*g/mL) in mesenteric rings pre-contracted with phenylephrine (1 *μ*M) in the presence (●) or absence (○) of functional endothelium. Results were expressed as mean ± SEM (n = 7 e 6, respectively).

After contraction induced by Phe (1 *μ*M); JSJ (10 - 5000 *μ*g/mL) induced concentration-dependent vasorelaxation in isolated rat superior mesenteric artery rings with endothelium (MR = 105.3 ± 3.54%, EC_50_ = 1172.7 ± 116.1 *μ*g/ml) (Figures 3(a) and 3(c)). Removal of the endothelium did not affect the JSJ-induced relaxant response, suggesting that JSJ exerts its effects through endothelial independent mechanisms (Figures 3(b) and 3(c)). It is important to point out that all effects induced by JSJ were completely reversible.

### 3.4. Effect of JSJ on Superior Mesenteric Artery Rings Pre-Contracted with Depolarizing K^+^ Solutions (KCl 60 mM)

**Figure 4:**
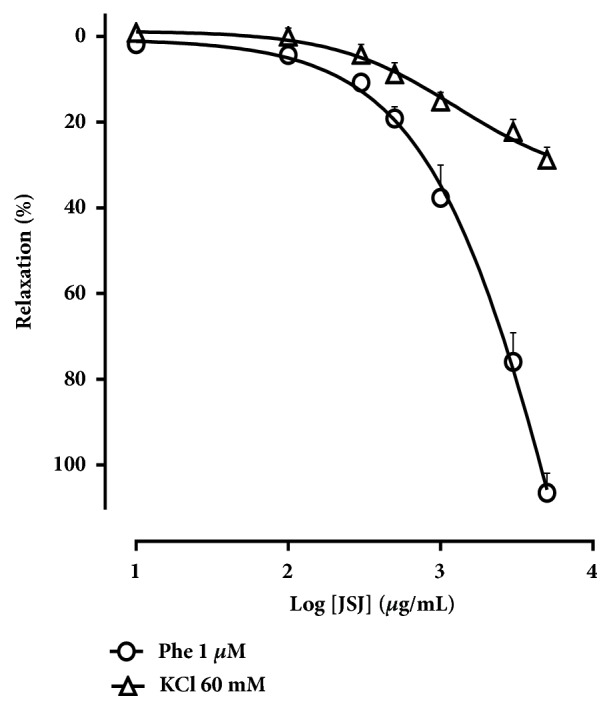
Concentration-response curves to JSJ (10 - 5000 *μ*g/mL) in mesenteric rings isolated rat pre-contracted with phenylephrine (1 *μ*M) (○) or KCl 60 mM (Δ) in the absence of functional endothelium. Results were expressed as mean ± SEM.

The JSJ induced vasorelaxation mechanism was investigated in pretreated (KCl 60 mM) endothelium-denuded mesenteric rings. In these preparations, JSJ relaxing activity was strongly inhibited (MR = 28.7 ± 2.8%) (Figure 4).

### 3.5. Involvement of K^+^ Channels in JSJ Induced Vasorelaxant Effect

**Figure 5:**
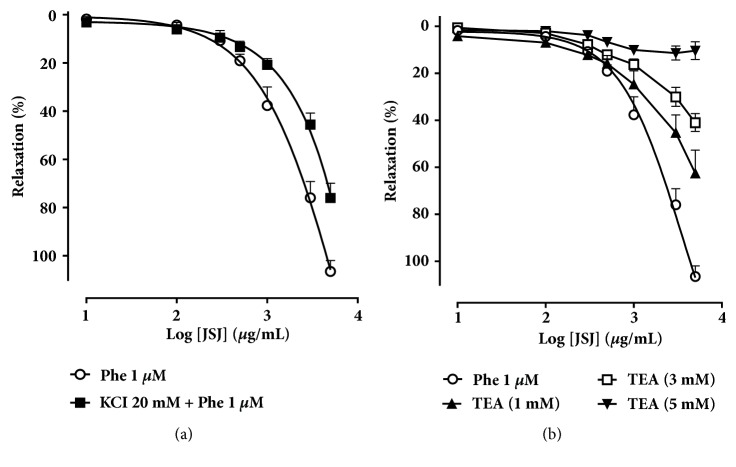
**(a)** Vasorelaxant effect induced by JSJ (10 - 5000 *μ*g/mL) in rings of the artery mesenteric artery isolated rat without endothelium contracted with Phe (1 *μ*M) (○, n=6) or in contracted with Phe in the presence KCl 20 mM (■) (n=5).** (b) **Contracted with Phe (1 *μ*M) (○, n=6) or contracted with Phe (1 *μ*M) in the presence of TEA (1 mM) (▲, n=8), TEA (3 mM) (□, n=7) or TEA (5 mM) (▼, n=5). Results were expressed as mean ± SEM.

The vasorelaxant effect induced by JSJ was inhibited in mesenteric rings pre-contracted with Phe (1 *μ*M) in the presence of Tyrode's solution containing 20 mmol/L KCl solution (MR = 75.9 ± 6.0%) as compared to the control (MR = 106.4 ± 4.5%) (Figure 5(a)). This suggested the involvement of K^+^ channels in the JSJ vasorelaxant effect. In the preparations incubated with different TEA concentrations (1, 3 and 5 mM), a K^+^ channel blocker, we observed significant attenuation in the concentration-response curve produced by JSJ. The effect was concentration-dependent (MR = 62.5 ± 9.8%, 40.9 ± 3.8% and 10.3 ± 3.7%, respectively) (Figure 5(b)). Interestingly, the effect was essentially abolished in the presence of TEA (5 mM).

### 3.6. Participation of K^+^ Channels Subtype in the JSJ-Induced Vasorelaxation

**Figure 6:**
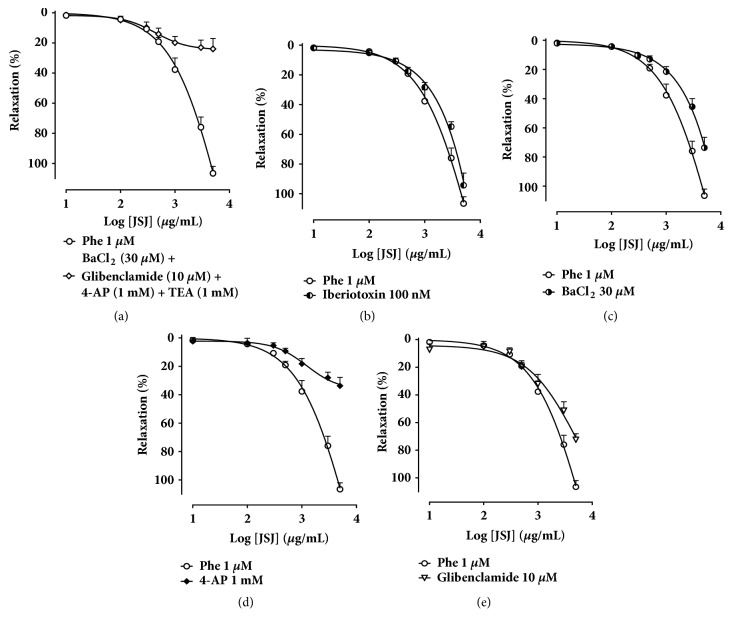
Concentration-response curves showing the participation of K^+^ channels in the vasorelaxant effect induced by JSJ.** (a) **Relaxation induced by JSJ in the endothelium-denuded mesenteric artery rings pre-contracted with Phe (1 *μ*M) in the absence (○; n=6) or in the simultaneous presence of potassium channel blockers for 4-AP (1 mM), glibenclamide (10 *μ*M), BaCl_2_ (30 *μ*M) and TEA (1 mM) (*◇*, n=5), or** (b) **Iberiotoxin 100 nM (◐, n=6), or** (c) **BaCl_2_ (30 *μ*M) (◑, n=7), or** (d) **4-AP 1 mM (◆), (n = 5), or** (e)** Glibenclamide 10 *μ*M (*∇*, n=7). The Values were expressed as mean ± SEM.

The effect of JSJ was also evaluated using 4-AP (1 mM), glibenclamide (10 *μ*M), BaCl_2_ (30 *μ*M), and TEA (1 mM), simultaneously. Its vasorelaxant effect was significantly attenuated (MR = 23.9 ± 3.4%) (Figure 6(a)). Iberiotoxin (100 nM) did not affect JSJ-induced relaxation (MR = 94.2 ± 8.1%, EC_50_ = 1735.0 ± 181.8 *μ*g/ml) in comparison with the control (MR = 106.4 ± 4.5%, EC_50_ = 1506.5 ± 148.1 *μ*g/ml) (Figure 6(b)). In the presence of BaCl_2_ (30 *μ*M) (MR = 73.5 ± 6.9%) (Figure 6(c)), the vasorelaxant effect induced by JSJ was significantly reduced. In the presence of 4-AP (1 mM) the relaxing activity of JSJ was strongly inhibited (MR = 33.6 ± 5.9%) (Figure 6(d)). In addition, glibenclamide (10 *μ*M) (MR = 72.3 ± 4.3%) (Figure 6(e)) also induced significant reduction in the JSJ effect.

### 3.7. Effect of JSJ on the Cumulative Curve for CaCl_2_ in Mesenteric Rat Arteries

**Figure 7:**
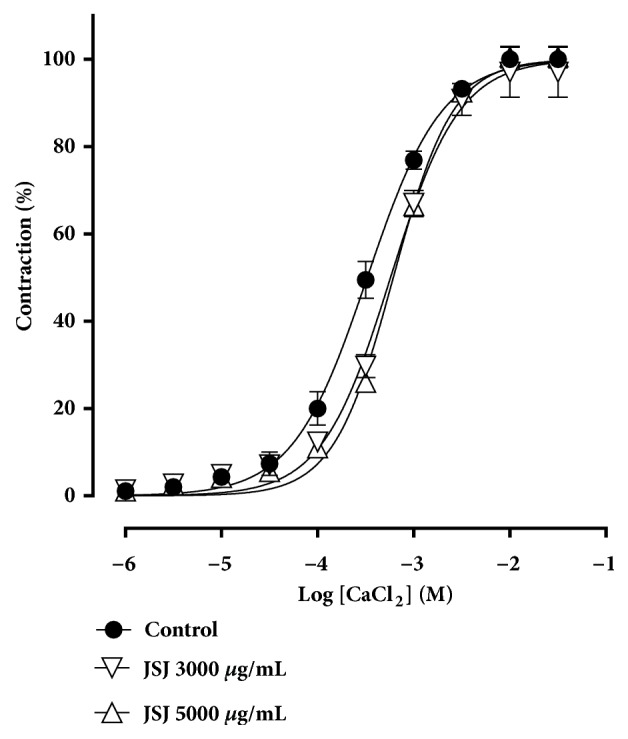
Inhibitory effect of JSJ on CaCl_2_ induced contractile response in endothelium-denuded mesenteric rings. Concentration-response curves for CaCl_2_ were determined in the absence (control) and after the incubation with JSJ at 3000 or 5000 *μ*g/mL (n = 5). The values were expressed as mean ± S.E.M.

Figure 7 shows the concentration-response curves for CaCl_2_ presenting no change in the maximum JSJ response. However, there was a slight displacement of the curves to the right, changing its potency. The values obtained in these experimental conditions were as follows: MR = 97.05 ± 5.71%; pD_2_ = 3.25 ± 0.03; n = 4; and MR = 100.51 ± 2.46%; pD_2_ = 3.19 ± 0.01; n = 4, for the respective concentrations of 3000 and 5000 *μ*g/mL. These values were compared with those obtained in the controls MR = 100 ± 0.00%; pD_2_ = 3.47 ± 0.02; n = 4.

### 3.8. Effect of JSJ on K^+^ Current in Vascular Myocytes

**Figure 8:**
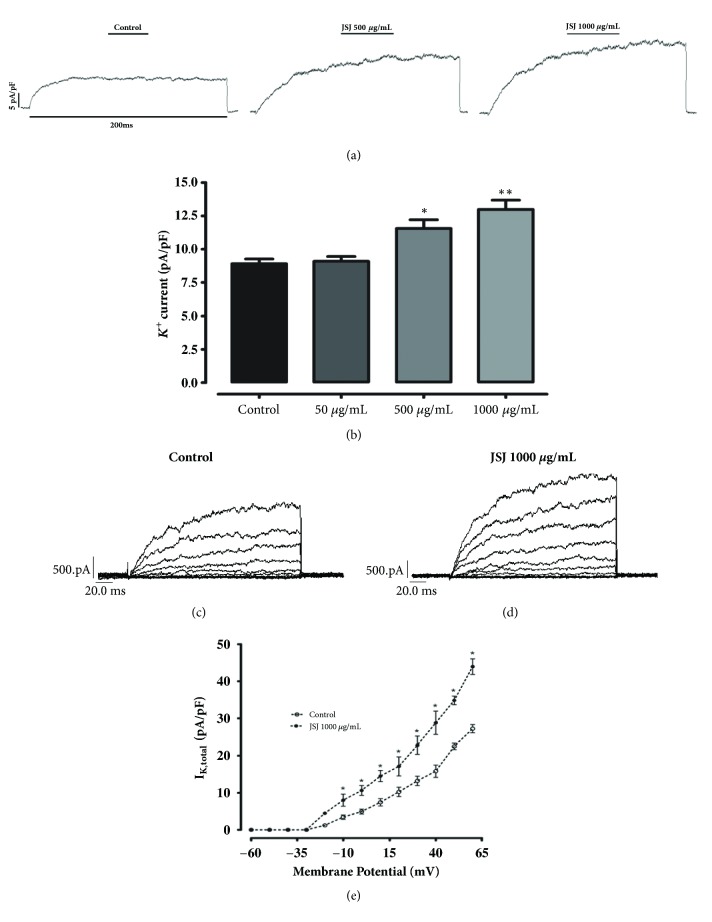
Effect of JSJ on potassium currents in mesenteric smooth muscle cells.** (a) **Representative IK recordings before (control) and after JSJ perfusion at 500 *μ*g/mL and 1000 *μ*g/mL. Currents were elicited by depolarizing pulses to +60 mV at 200 ms duration from a holding potential of -60 mV.** (b) **Bar plot showing statistical analysis obtained from the maximum value of current efflux (pA/pF) at each differing JSJ concentration. Control was absent of JSJ perfusion.** (c) **Representative recordings of IK total acquired without JSJ incubation.** (d) **IK recordings displayed for JSJ at 1000 *μ*g/mL. The recordings were obtained by triggering depolarizing pulses from -60 mV to + 60 mV in 10 mV steps. The holding potential was set at -60 mV.** (e) **I-V relationship of IK total in the absence (open circles) or presence (filled circles) of 1000 *μ*g/mL JSJ perfusion. Results represent the mean ± SEM; (n=7; *∗*p<0.05; *∗∗*p<0.01).

To directly confirm the effect of JSJ stimulation in vascular smooth muscle potassium channels, total IK concentration-response relationships in mesenteric myocytes were tested.  Figure 8(a) presents typical current recordings without JSJ incubation (control) and in the presence of JSJ infusions at 500 and 1000 *μ*g/mL.  Figure 8(b) summarizes data, showing the concentration-response relationship for potassium channel activation where JSJ increased total IK of 8.90 ± 0.35 pA/pF (control) to 9.09 ± 0.36 pA/pF, 11.56 ± 0.65 pA/pF, and 12.99 ± 0.69 pA/pF, for respective JSJ perfusions of 50, 500 and 1000 *μ*g/mL. To better show the current increases caused by JSJ, current-voltage ratios were examined. A typical family of current (control) traces is shown in Figure 8(c). Recordings after treatment with 1000 *μ*g/mL of JSJ are presented in Figure 8(d). JSJ (1000 *μ*g/mL) caused an increase in total IK (Figure 8(e)), which is seen in the most depolarizing pulses. This is because there was a significant increase in potassium current, from -10 mV to the most depolarizing pulse tested.

## 4. Discussion

The present study increases our knowledge concerning the JSJ mechanism of action on the cardiovascular system. Acute administration of JSJ promoted hypotensive effect related to reductions in total peripheral vascular resistance, as was observed in the vasorelaxant effect produced in mesenteric rat artery rings. The mechanism likely involves activation of K^+^ channels, and consequent membrane hyperpolarization in vascular smooth muscle cells.

Preliminary JSJ phytochemical analysis revealed the presence of flavonoids and steroids. The results corroborate data obtained by Pereira (2011) showing the presence of flavonoids in aqueous* Syzygium jambolanum* (Lam.) DC bark and pulp extract [18]; and also Faria et al. (2011) [19], who detected the presence of steroids in aqueous extracts of the pulp. However, the alkaloids and tannins found in the literature were not detected [19]. Phenolic compounds are major antioxidants found in plants and medicinal herbs. The HPLC analysis indicates that there were significant amounts of phenolic compounds in the JSJ extract and that 2,5-dihydroxybenzoic and caffeic acid were present in larger quantities in the JSJ.

Vasco, Ruales, and Kamal-Eldin (2008) [20] analyzed the total phenolic content of seventeen fruits from Ecuador, the fruits were assessed for phenolic compounds in fresh samples in three categories: low (<100 mg GAE/100 g, medium (100 to 500 mg GAE/100 g) and high (> 500 mg GAE/100 g) [20]. The total phenolic content presented by JSJ was 988.55 ± 5.41 (mg GAE/100g), thus containing a high total content of phenols. This result corroborates studies conducted by Maria Do Socorro et al. (2010) that showed a polyphenol content of 1117 ± 67.1 (mg GAE/100g) [21]. The antioxidant activity presented by JSJ, expressed as EC_50_, yielded little capacity to chelate the DPPH radical●. This corroborated the data presented by Reynertson et al. (2008), which yielded 389 ± 36.0 *μ*g/ml [22].

Several foods rich in polyphenols, for example, red wine, chocolate, green tea, fruits, and vegetables have demonstrated the ability to reduce the risk of cardiovascular diseases [22, 23]. Assessment of the JSJ response induced on blood pressure and heart rate was performed in non-anesthetized normotensive rats. Acute administration of JSJ (i.v.) promoted hypotension followed by tachycardia. Studies performed with hydroalcoholic extract from* Syzygium jambolanum* fruit also demonstrated hypotensive activity in normotensive and spontaneously hypertensive rats [7, 8].

In order to understand the mechanism of JSJ-mediated hypotension and bearing in mind that a reduction in peripheral vascular resistance causes a decrease in the blood pressure, we hypothesized that JSJ could probably act by relaxing the vascular tissue and thus decreasing peripheral vascular resistances in rat superior mesenteric arteries. Using Phe (1 *μ*M), a contracting agent, we evaluated the effect of JSJ facing preparations with contracted superior mesenteric artery rings. The results showed that JSJ induces concentration-independent relaxation of the vascular endothelium. Taken together these results are in agreement with findings in the literature [7, 8]. In addition, we can hypothesize that the hypotensive and vasorelaxant effects induced by JSJ can be attributed to its high levels of phenolic content.

Substances with vasorelaxant action may promote the response by inducing relaxation of vascular smooth muscle through direct activity in vascular smooth muscle cells, or in endothelial cells which in turn regulate vascular smooth muscle cell contraction. Our results suggest that JSJ exerts its effect on vascular smooth muscle cells. From these preliminary results, subsequent experiments were performed with mesenteric artery rings without endothelium and submitted to precontractions.

It is well known that phenylephrine induced vasoconstriction is mediated by stimulation of alpha-adrenergic receptors coupled to G proteins. KCl induces smooth muscle contraction by decreasing K^+^ efflux, promoting depolarization, and consequent opening of voltage-dependent Ca^2+^channels (Ca_V_) [24, 25]. Thus, we sought to evaluate the effects of JSJ on mesenteric artery rings when contracted with depolarizing solution containing 60 mM KCl. Under these conditions, the vasorelaxation effect induced by JSJ was markedly reduced as compared to that obtained for mesenteric artery rings precontracted with Phe (1 *μ*M).

In the literature, due to the decrease in K^+^ efflux, drugs that promote relaxation by activation of potassium channels present reduced activity against contractions induced by depolarizing agents [26]. Thus, our results suggest that the vasorelaxation promoted by JSJ may involve the activation of K^+^ channels. Based on this, and the importance of K^+^ channels in regulating vascular functions, we evaluated the participation of these channels in JSJ induced vasorelaxant response. For this we used Tyrode's solution modified with 20 mM KCl, a concentration sufficient to partially prevent efflux of K^+^ and attenuate vasorelaxation mediated by the opening of K^+^ channels [16, 17]. Additionally, we also experimented using TEA, a blocker of K^+^ channels, at different concentrations (1, 3, and 5 mM) [27–29]. In all these situations, the effect of JSJ was significantly attenuated, and, for the differing TEA concentrations, the effect was concentration-dependent. These data suggest the involvement of K^+^ channels in the vasorelaxant effect induced by JSJ.

Activation of these channels promotes an increase in K^+^ efflux producing hyperpolarization of vascular smooth muscle. The activity of potassium channels plays an essential role in regulating the membrane potential and vascular tonus [30]. Changes in the expression and function of K^+^ channels have been observed in cardiovascular disorders [31]. Data reported in the literature suggest the existence of different K^+^ channel subtypes expressed in the membrane of vascular smooth muscle cells. Four distinct subgroups of these channels have been identified in arterial smooth muscle: K^+^ channels dependent on voltage (K_V_); K^+^ channels sensitive to ATP (K_ATP_); K^+^ input rectifier channels (K_IR_); and large conductance K^+^ channels sensitive to Ca^2+^ (BK_Ca_) [32].

Thus, we evaluated which subtypes of potassium channels are involved in the JSJ induced vasorelaxant response. Initially we used differing potassium channel blockers simultaneously and observed that the JSJ concentration-response was markedly attenuated, with a 23% residual relaxation. The relaxing effect of JSJ was also inhibited by the isolated presence of BaCl_2_, glibenclamide, and 4-AP. However, incubation with iberiotoxin did not change the maximum effect or potency. The results together show the involvement of three potassium channels subtypes: K_IR_, K_ATP_, and K_V_ in the JSJ induced vasorelaxant, mainly, K_V_. To further confirm that K^+^ channel activation is definitely involved the vasorelaxant effect of JSJ, we used patch-clamp whole-cell technique. The results demonstrated that JSJ increases K^+^ currents in isolated smooth muscle cells from mesenteric arteries, thus confirming our hypothesis that the activation of K^+^ current contributes to JSJ-induced relaxation.

Studies show that vascular smooth muscle cells contractility can be regulated by the intracellular calcium concentration ([Ca^2+^]_i_), with entry of Ca^2+^, associated with [Ca^2+^]_i_ increases, facilitation of (Ca^2+^) 4-CaM complex (calmodulin) interactions (which after undergoing conformational change), activating myosin light chain kinase, which phosphorylates myosin light chain, favoring actin filament sliding over myosin, and consequently generating contraction force in smooth muscles [33]. The literature reports that a large number of substances derived from medicinal plants (including* Syzygium jambolanum* hydroalcoholic leaf extract) act by modulating smooth muscle cell Ca^2+^ channels [3].

Based on these reports, we sought to observe if the vasorelaxant effect induced by JSJ was related to inhibition of Ca^2+^ influx via Ca_v_. We investigated the effect of JSJ on contractions induced by CaCl_2_, in a depolarizing medium, nominally without calcium. Under these conditions, JSJ did not alter the maximum effects of contractions induced by CaCl_2_. However, there was a slight displacement of the curves to the right, indicating changing potency. This suggests that a small part of the vasorelaxant effect induced by JSJ may be related to its influence on Ca_v_ channels, resulting in a decrease of Ca^2+^ influx in superior mesenteric rat artery smooth muscle and consequently in vasodilation. Thus, we can hypothesize that Ca_v_ channel blockade may be the mechanism of the residual relaxation, in approximately 24%, observed after potassium channel blockers mixture incubation.

## 5. Conclusions

Thus, considering the results obtained in this study, we conclude that JSJ is able to promote hypotension in rats and vasorelaxation in rat mesenteric artery rings. The vasorelaxant action induced by JSJ may involves, at least, the activation of potassium channels: K_ATP_, K_v_, and K_ir_ channels. Furthermore, the results obtained so far corroborate the therapeutic potential of the* Syzygium jambolanum* (Lam.) DC for the treatment of cardiovascular disorders.

## Figures and Tables

**Table 1:** Phenolic compounds detected in JSJ by HPLC.

**Phenolic compounds**	**Concentration of the compound** **mg/100 g of JSJ**
2,5-dihydroxybenzoic acid	0.8186
Caffeic acid	0.6549
Vanillic acid	0.4093
Rutin	0.3274
Quercetin	0.2456
Chrysin	0.2047
Catechin	0.1228
4-hydroxybenzoic acid	0.1226
Myricetin	0.1226
p-Coumaric acid	0.0409
Syringic acid	0.0408

## Data Availability

The data used to support the findings of this study are available from the corresponding author upon request.
